# Collagen Biomarkers for Arthritis Applications

**Published:** 2007-02-07

**Authors:** James D. Birmingham, Vladimir Vilim, Virginia B. Kraus

**Affiliations:** 1 Duke University Medical Center, Durham, North Carolina, USA; 2 Institute of Rheumatology, Prague, Czech Republic

**Keywords:** type II collagen, biomarkers, osteoarthritis, rheumatoid arthritis, ankylosing spondylitis

## Background

The most common form of chronic arthritis is osteoarthritis (OA) with prevalence as high as 80% after age 75 (Arden and Nevitt, 2006). The incidence of OA is expected to increase as the population ages, increasing the socioeconomic burden of OA. Despite the significant burden of this disease, no drug has been identified that can effectively modify disease progression (Moskowitz and Hooper, 2005; Abadie et al. 2004). However, slowing disease progress and improvement in quality of life may be achieved by behavioral modifications, such as weight loss and exercise. Many patients with early OA will progress to disability and joint replacement. Physical examination and radiographic studies are relatively poor means for detecting disease early or predicting progression. Therefore, identification of factors to facilitate early OA diagnosis and prognosis is a major focus of current OA research (Lohmander and Felson, 2004; Lohmander, 2004; Garnero and Delmas, 2003).

Considerable intellectual and financial resources are currently being invested into this biomarker development effort worldwide in the hope that biomarkers will help in the following ways: i) to diagnose OA prior to the stage when loss of articular cartilage can be measured on a radiograph, ii) to identify patients with increased risk of progression, iii) to monitor effectiveness of therapeutic interventions, and iv) to select patients for clinical trials of new drugs. To this end, numerous potential OA biomarkers have emerged over the last decade (Moskowitz and Hooper, 2005). These biomarkers comprise a diverse group of molecules derived from all components of the joint. Moreover, studies of these molecules have provided valuable insights into cartilage pathobiology (Poole, 2003).

An imbalance in cartilage synthesis and degradation is central to the development of OA (Guilak et al. 2004; Lippiello et al. 1977; Malemud et al. 2003). Therefore, products of cartilage metabolism have been explored as potential candidate biomarkers for OA. Cartilage is composed of two major proteins, aggrecan, and type II collagen, in addition to many less abundant proteins such as decorin, fibromodulin, cartilage oligomeric matrix protein (COMP), cartilage intermediate layer protein (CILP), proline arginine-rich end leucine-rich protein (PRELP), various minor collagens, link protein, fibronectin, and the glycosaminoglycan- hyaluronan (Saxne, 2006). Potential OA related biomarkers can originate both within and outside of joint tissues (Table 1). The focus of this review is type II collagen, the most abundant protein component of cartilage and a molecule that has yielded a wealth of potential OA-related biomarkers.

Many different type II collagen epitopes have been described as potential OA biomarkers (Table 2). Interestingly, many of these epitopes can reflect different biological processes even though they originate from the same molecule. For instance, collagen provides epitopes indicative of both degradative or catabolic events, and synthetic or anabolic events. Simultaneous assessment of both collagen degradation and synthesis in a patient is a particularly promising approach for diagnosing and determining risk of OA progression (Poole, 2003). Thus, type II collagen serves as a useful and instructive paradigm of the biomarker development process. In conjunction with the presentation of the human clinical evidence available for each of the collagen biomarkers, we make use of the newly proposed BIPED classification scheme put forth by the Osteoarthritis Biomarkers Network (Bauer et al. 2006). The proposed algorithm classifies markers into five categories for the purpose of applying a common biomarker vocabulary across investigators, their laboratories and potentially across fields, and to facilitate the design of biomarker validation studies. The BIPED acronym represents the following categories: burden of disease, investigative, prognostic, efficacy of intervention, and diagnostic; when possible, we have related the outcomes of the available relevant human clinical data to one or more of these categories.

## Collagen Biomarkers

### Collagen Synthesis and Degradation

Type II collagen is a homotrimer of a1(II) chains. These undergo hydroxylation, interchain disulfide bonding, and triple helix formation (winding from the C- toward the N-terminus). The molecule is then secreted to the extracellular matrix of cartilage. There are two procollagen variants of type II collagen derived by alternative splicing: procollagen IIA possessing a cysteine-rich von Willibrand factor C-like domain in the N-terminal propeptide (PIIANP) (O’Leary et al. 2004), and procollagen IIB lacking this cysteine-rich domain within the amino propeptide (McAlinden et al. 2005). The procollagen IIA variant is expressed during fetal development and during OA in what is believed to be an attempted repair response to cartilage degradation (Aigner et al. 1999; Salminen et al. 2001), procollagen IIB is expressed in normal adult cartilage. These procollagen forms undergo processing to the mature form with release of N- and C- terminal propeptides that are indicative of the amount of newly synthesized collagen. Groups of mature collagen fibrils associate in regular staggered arrays and undergo cross-linking to form larger fibrils and fibers (Gelse et al. 2003).

Type II collagen is cleaved by collagenases known as metalloproteinases (MMPs); MMP-1, 8, and 13 are thought to be particularly important. Collagenase mediated cleavage results in two fragments: a ¾ length fragment (also referred to as TC^A^) and a ¼ length fragment (TC^B^). This proteolysis causes a loss of type II collagen epitopes to body fluids wherein they can indicate the amount of degradation of collagen. The ability to monitor and slow or reverse this process has important clinical and therapeutic implications because extensive degradation of mature cross-linked type II collagen fibers is considered to be a critical and perhaps irreversible stage in joint destruction (Billinghurst et al. 1997; Nelson et al. 1998).

### Collagen Degradation Biomarkers

Type II collagen is possibly the ideal marker of cartilage degradation. First, it is relatively specific to articular cartilage, although it is also found in other cartilages as well as the vitreous humor of the eye and the nucleus pulposus of lumbar discs (Burgeson and Nimni, 1992; Elsaid and Chichester, 2006). Second, type II collagen is the most abundant protein in cartilage, representing 15–25% of the wet weight, 50% of the dry weight, and 90–95% of the total collagen content. Third, type II collagen turnover is normally very slow, with a biological half-life estimated to be 117 years in adult cartilage (Verzijl et al. 2000), thus pathological turnover is readily detected above background metabolism. Furthermore, many assays have been produced to measure the products of type II collagen metabolism in the serum, synovial fluid, and urine of animal models of arthritis as well as in humans. The type II collagen biomarkers indicative of degradation fall into three groups according to the localization of the particular epitope within the collagen molecule: cleavage neoepitopes localized to the collagenase cleavage site; denaturation neoepitopes localized to the triple-helical domain and made accessible to the detection reagents by unwinding of the triple helix; and epitopes localized to the mature ends or telopeptides of the molecule (Table 2).

#### Cleavage neoepitopes

The process of collagen cleavage and denaturation of the triple helical molecule exposes certain protein sequences, termed neoepitopes, within the collagen molecule. These neoepitopes can be measured and correlated with the amount of cartilage degradation.

##### Col 2¼– N1 and ¼N2

Billinghurst and Poole were the first to report on the use of antibodies to detect neoepitopes of collagen generated by collagenase cleavage (Billinghurst et al. 1997). COL2-1/4N1 is an amino-terminal neoepitope on the shorter fragment (TC^B^) of collagen, and COL2-1/4N2 is generated by a secondary collagenase cleavage resulting in an amino-terminal neoepitope lacking 3 amino acid residues of the TC^B^ fragment. Little else has been published on the use of these markers for OA diagnosis, progression, or response to therapy.

##### C_2_C and C_1,2_C

Neoepitopes identified at the C-terminus of the ¾ length fragment, include C_2_C and C_1,2_C. C_1,2_C is a carboxy-terminal cleavage neoepitope on the ¾ fragment of collagen. The name ‘C_1,2_’ signifies that this neoepitope is not specific for collagen type II but is also generated upon collagenase cleavage of type I collagen, exposing the 5 amino-terminal residues on the ¾ fragment that are identical for both collagens type I and II. C_1,2_C is the product of secondary cleavage of the C_2_C product (described below) and is resistant to further degradation. Mean (SD) serum values for non-arthritic controls are available in the literature only for females (314 ± 119 ng/ml) (Haima, 2005). This neoepitope is also referred to as COL2-3/4Cshort, signifying the length of the epitope, not the length of the fragment on which the epitope is located (Poole et al. 2004). This assay, which is ELISA based and commercially available (IBEX, Montreal, Quebec), can be used on serum, synovial fluid, and cartilage.

As a diagnostic marker, C_1,2_C has been shown to be elevated in cartilage from patients with OA compared to controls (17 pmole/mg cartilage compared to 7 pmole/mg; p value 0.0002) (Billinghurst et al. 1997). In a cartilage explant system, cleavage of type II collagen by collagenase was significantly increased in OA cartilage samples, generating higher levels of C_1,2_C in vitro from OA cartilage; this elevation could be blocked by collagenase inhibitors (Dahlberg et al. 2000). Collagenase inhibitors can also inhibit the loss of this epitope from bovine cartilage explants in response to IL-1 (Billinghurst et al. 2000). In a population-based sample in humans, serum C_1,2_C has been associated with incident radiographic knee OA (Jordan et al. 2004b) and is higher in African Americans than Caucasians (Jordan et al. 2004a).

C_2_C is measured by a commercially available ELISA (IBEX, Montreal, Quebec). The epitope is located at the C-terminus of the ¾ length fragment. The problem of specificity for type II collagen posed by C_1,2_C was solved by lengthening the C_1,2_C epitope sequence by three amino acid residues (GGEGPP(OH)GPQG) where E corresponds to a residue specific to human type II collagen, and the tandem GG represents a spacer, thought to be important for recapitulating a specific conformation of the native epitope upon release from the triple helix. This neoepitope, originally designated COL2-3/4C_long_, is now known as C_2_C (Poole et al. 2004). Hydroxylation of P_902_ within this epitope is essential for C_2_C immunoreactivity while hydroxylation at other sites within this epitope abolish C_2_C immunoreactivity (Poole et al. 2004). Therefore, subtle species differences in hydroxylation of this sequence may dramatically impact the ability to detect this epitope with the anti-C_2_C monoclonal antibody. As with C_1,2_C, mean serum values are available in the literature only for non-arthritic females (58 ± 28 ng/ml) (Haima, 2005). This assay can be used on serum, urine, or synovial fluid in humans (Fraser et al. 2003; Cibere et al. 2005), and animals (Kojima et al. 2001; Song et al. 1999; Chu et al. 2002), although the specificity of the assay for type II collagen in animals is in question due to the fact that residue E_899_ of the C_2_C epitope, corresponding to human type II collagen, is a D in monkeys, rodents, including guinea pigs, horses, dogs and chickens. Levels of this neoepitope were high a few weeks after an experimentally induced inflammatory arthritis (collagen induced arthritis, CIA) or OA (Song et al. 1999; Kojima et al. 2001; Chu et al. 2002; Kojima, 2004; Billinghurst et al. 2001). This neoepitope has declined in association with reduced destruction of joint cartilage in a rat model of CIA treated with collagenase inhibitors (Song et al. 1999). In a transgenic mouse model of OA, C_2_C was elevated before the onset of clinically apparent cartilage lesions (El-Maadawy et al. 2003). These animal studies establish the utility of this assay for detection and quantification of cartilage degradation.

A significant correlation has been observed for serum C_2_C and cartilage volume by magnetic resonance imaging in patients with symptomatic knee OA (King et al. 2004). C_2_C has also been associated with incident radiographic knee OA (Jordan et al. 2004b). Treatment of rheumatoid arthritis patients with infliximab and methotrexate resulted in reduction of serum C_2_C levels compared to levels in placebo treated patients and correlated with symptomatic improvement in the ASPIRE trial (Visvanathan et al. 2004). Compared to placebo, treatment of ankylosing spondylitis patients with etanercept resulted in suppressed serum C_2_C in a manner that correlated with the decline in sedimentation rate (ESR) and C-reactive protein levels (Maksymowych et al. 2005). However, no difference in urinary or serum C_2_C, C_1,2_C, or their ratio were observed between groups following treatment for 6 months with glucosamine or placebo for knee OA (Cibere et al. 2005).

The combination of C_2_C with C_1,2_C may improve the ability to predict which patients will have progressive OA. In a study of 209 patients with established knee OA, higher ratios of C_1,2_C/C_2_C were observed for OA progressors compared to non-progressors over 18 months (Cerejo et al. 2002; Sharma et al. 2004). They speculated that the higher ratio in progressors was due to increased secondary cleavage of the long (¾) fragment of type II collagen. They have reported an increased odds of OA progression for the highest tertiles of C_2_C:CPII ratios (Odds Ratio 3.15), and C_1,2_C:CPII ratios (Odds Ratio 1.79). In a cohort of 330 individuals in a population based study, the C_2_C/C_1,2_C ratio was also associated with knee OA progression (p = 0.06) (Jordan et al. 2004b). Overall, these studies support the designation of C_2_C, or C_2_C in combination with C_1,2_C or CPII, for the diagnosis and prognosis of OA and for monitoring the efficacy of some therapeutic interventions for OA.

##### uTIINE

The urinary Type II Collagen NeoEpitope (uTIINE) is detected by a sandwich ELISA with specificity for type II collagen degradation. The detection monoclonal antibody (mAb) used in this assay, 9A4, binds the type II collagen neoepitope generated at the new C-terminus on the collagen ¾ fragment following collagen cleavage and detects the same collagen neoepitope as C_1,2_C (Otterness et al. 1999). Specificity of the assay for type II collagen is achieved with the capture mAb 5109 (Otterness et al. 1999; Downs et al. 2001), which is only capable of binding type II collagen in monomeric as opposed to native triple helical collagen conformation. Three amino acid residues (GAE) separate the epitopes for these two mAbs, preventing steric competition. Recently a liquid chromatography-mass spectrometry/mass spectroscopy (LC-MS/MS) format of the assay has been developed that relies on immunoaffinity capture with the 5109 antibody. This assay has led to the demonstration that a 45-mer peptide is the most abundant uTIINE species in the urine and synovial fluid (Hellio Le Graverand et al. 2006). Of note, this assay is not useful in rabbits and guinea pigs because of sequence differences in the epitope recognized by the antibody 5109. In principle, an epitope present in serum has to be present also in synovial fluid, and an epitope present in urine has to be present also in serum and synovial fluid. However, in the case of uTIINE, this urinary epitope is not found in serum at the level of detection of the assay (0.169 ng/ml, 0.05 nM). This has been ascribed to lack of reabsorption into the plasma after glomerular filtration due to the size and chemical properties (highly polar and acidic) of this peptide (Hellio Le Graverand et al. 2006).

Early investigative work showed that measurable quantities of uTIINE were detectable in 9/10 OA patients but only 2/10 controls (Downs et al. 2001). In this study, the uTIINE concentrations in OA patients were almost three-fold higher than the 2 controls with the measurable levels (312 pM and 123 pM respectively) (Downs et al. 2001). These data support the use of this ELISA for the diagnosis of OA. OA and RA severity have also been assessed with uTIINE and levels were found to correlate with and predict disease status in RA (Saltarelli et al. 1999; Woodworth et al. 1999). Levels of uTIINE do not vary diurnally and decreased in response to initiation of methotrexate therapy in newly diagnosed rheumatoid arthritis patients (Pickering et al. 2000; Saltarelli et al. 2000). Using the new LC-MS/MS format in a cross-sectional study, uTIINE was ~50% higher in individuals with symptomatic radiographic OA of the hip or knee compared to individuals with asymptomatic radiographic knee OA, and individuals over age 55 years without radiographic OA (Pickering et al. 2004). Again using this newer assay format, serial uTIINE concentrations reflected concurrent joint space narrowing in a trial evaluating the effect of doxycycline for knee OA (Hellio Le Graverand et al. 2006) suggesting a possible role as a burden of disease marker. Levels of uTIINE have also been shown to reflect cartilage degradation in relapsing polychondritis (Kraus et al. 2003), a severe autoimmune disorder associated with hyaline cartilage destruction. In this study, uTIINE levels were elevated prior to treatment, fell with anti-tumor necrosis alpha therapy in concert with symptomatic improvement, and then rose again after therapy was discontinued. These studies provide a rationale for the use of this ELISA to follow type II collagen degradation in response to therapy.

#### Collagen denaturation epitopes

After the initial cleavage of the collagen triple helix by collagenase, the helix can denature into the monomeric *α*1(II) chains. Denaturation epitopes are “unmasked” and made accessible following the unwinding of *α*1 chains from the usual conformational restriction in the triple-helix.

##### Col2-3/4m

Dodge and Poole used denatured (unwound) *α*-chains of cyanogens bromide (CNBr)-derived peptides of bovine tropocollagen type II as immunogens (*α*1(II)CB11 - residues 254–533, and *α*1(II)CB8 - residues 533–682 of Figure 1a) to prepare polyclonal antiserum R181 (Dodge and Poole, 1989). These CNBr peptides were localized to the first (N-terminal) half of the helical domain and did not contain the collagenase cleavage site. The antiserum did not react with native type II collagen or collagens of other types and was used in immunohistochemical studies (Dodge and Poole, 1989; Dodge et al. 1991). One of five hydrophilic domains from the CNBr-derived peptide, *α*1(II)CB11, was subsequently synthesized and used to prepare a monoclonal antibody (mAb) designated COL2-3/4m, referring to the fact that the epitope is localized to the larger of the two collagenase cleavage fragments of collagen (Hollander et al. 1994). The antibody does not interact with native type II collagen but there is activity against homologous sequence in type XI collagen [*α*3(XI) chain], which is present in very small amounts in cartilage. This mAb has also been used preferentially in immunohistochemistry studies demonstrating damage of type II collagen in human articular cartilage in OA (Hollander et al. 1994; Hollander et al. 1995). Increased levels of COL2-3/4m are found in superficial layers of OA cartilage early in the disease process and increased levels in deeper layers as the disease progresses (Hollander et al. 1995).

Investigational use of this marker has demonstrated its utility for identifying cartilage damage in rodent models of arthritis. COL2-3/4m was elevated in the knees of experimental mouse models of arthritis, particularly in fibrillated areas compared to non-arthritic control knees (Stoop et al. 1999b). This elevation began within 3 days of experimental arthritis and remained elevated at 28 days. The same investigators found that COL2-3/4m was able to identify cartilage denaturation in a mouse model of spontaneous OA as well as an injury model of experimental OA in rats (Stoop et al. 1999a; Stoop et al. 2001). In human studies, this assay has shown potential as a diagnostic marker by differentiating OA from non-OA with 6-fold more denatured type II collagen in OA samples versus controls (Hollander et al. 1994). Data are lacking as of yet to support the classification of this marker as a burden of disease, prognostic, or efficacy of intervention marker.

##### Coll 2-1 and Coll 2-1 NO_2_

Henroitin et al. prepared rabbit antisera to a denaturation neoepitope, Coll 2-1, that corresponds to a peptide localized to another part of the helical domain of type II collagen (Deberg et al. 2002; Henrotin et al. 2004). Since the sequence contains tyrosine, an analogous competition ELISA was developed for the nitrated tyrosine form of the peptide (Coll 2-1 NO_2_). Using antisera (D3 and D37) derived from rabbits, the mean serum levels of these epitopes in adults aged 20–65 years were 125.13 ± 3.71 nmol/l (Coll 2-1), and 0.16 ± 0.08 nmol/l (Coll 2-1 NO_2_), and did not vary by age. They demonstrated higher levels of Coll 2-1 NO_2_ in females under the age of 45 compared with men (Henrotin et al. 2004).

Coll 2-1 is significantly elevated in OA patients compared to control (267.45 ± 26.42 nmol/l versus 126.78 ± 6.61 nmol/l) (Henrotin et al. 2004). Additionally, the ratio of Coll 2-1 NO_2_ to Coll 2-1 was shown to differentiate RA from OA with a 1.6 fold higher ratio in RA compared to OA (p < 0.05) (Deberg et al. 2005a). Higher levels of Coll 2-1 and Coll 2-1 NO_2_ in the urine have been shown to predict progression of radiographic joint space narrowing over one year (Henrotin et al. 2004; Deberg et al. 2005b). However, no association was found between these marker levels and radiographic grade of severity of joint disease. Therefore, these markers are not yet able to claim utility in burden of disease assessments. Data are not available to assess their ability to serve as indicators of the efficacy of therapeutic interventions.

##### HELIX-II

HELIX-II is detected in urine by a competitive ELISA using polyclonal antisera. This degradation epitope of the helical region of type II collagen was recently quantified in patients with OA, RA, and Paget’s disease, and healthy controls (Charni et al. 2005). The HELIX-II ELISA showed no significant cross-reactivity with human intact or denatured type II collagen, with similar peptides from human type I or type III collagens, or with elongated or shortened HELIX-II peptides, indicating that the HELIX-II ELISA recognizes a neoepitope from the alpha 1 chain of type II collagen that is unmasked upon collagen denaturation (Charni et al. 2005). As a diagnostic marker, HELIX-II concentrations were significantly higher in patients with OA (by 56%, P < 0.0001) and RA (by 123%, P < 0.0001) (Charni et al. 2005) compared to healthy age- and sex-matched controls. In the same study, elevated HELIX-II was a risk factor for radiographic damage in RA, implying utility as a prognostic marker. This effect was found to be independent of C-reactive protein, baseline damage, and uCTX-II level (described below). Data on response to therapy are not available for this marker.

##### AH12, AH8 and AH9

The antibodies AH12 and AH8 were developed and applied in a sandwich ELISA patented by Hollander and Croucher (Hollander and Croucher, 1998). This assay detects two sequential epitopes separated by six amino acid residues in the amino-terminal portion of the triple helical domain of type II collagen, made accessible by unwinding of the triple helix. This assay is investigative as no published data exist on its utility in diagnosis, prognosis, or effect of therapy in humans. A third antibody, AH9, has been developed to a carboxy-terminal sequence within the triple helical domain of type II collagen. Together with AH9, the sandwich ELISA assay has been used to monitor the release of amino- and carboxy-terminal fragments of type II collagen from cartilage explants treated with IL-1alpha (Croucher and Hollander, 1999). Although they were readily able to detect AH12-AH8 reactive epitopes, they were unable to detect AH9 reactive epitopes. They suggested that that the amino-terminus is relatively resistant to further degradation while the carboxy-terminus is labile after the helical domain of type II collagen is denatured (Croucher and Hollander, 1999). These results demonstrate that the helical region of type II collagen is not uniform in its susceptibility to proteolysis. They concluded that this fact has important implications for the choice of epitopes that are likely to be good markers of damage to cartilage collagen.

##### CII CNBr9.7

Barrach and Chichester et al. have developed a sandwich ELISA assay specific for CNBr9.7, a cyanogen bromide peptide in the ¼ length cleavage fragment of type II collagen (Elsaid and Chichester, 2006). The capture antibody of this assay (18:6:D6) is specific to type II collagen while the detection antibody (14:7:D8) reacts to homologous sequence within types I, II, II and V collagen, although the authors also report some reactivity of both antibodies to sequences within CNBr11 in the ¾ length fragment of type II collagen (residues 254–533 of Figure 1a) (Elsaid and Chichester, 2006). They have characterized levels of CII CNBr9.7 in the synovial fluid of patients after acute knee injury or with OA and RA (Barrach et al. 1996; Elsaid et al. 2003), and in the synovial fluid of rabbits following meniscectomy (Felice et al. 1999). Synovial fluid concentrations of the CII epitope increased with severity of articular cartilage degradation (Barrach et al. 1996). Interestingly, the CII peptide concentrations were highest in synovial fluid from patients with acute knee injury (mean 0.94 *μ*g/ml) compared to synovial fluid from patients with OA (mean 0.13 *μ*g/ml), RA (mean 0.17 *μ*g/ml), or synovial fluid from normal knees obtained postmortem (undetectable at < 50 ng/ml) (Elsaid et al. 2003).

#### Collagen telopeptides

##### Col2CTx and CTXII

**Figure 2:**
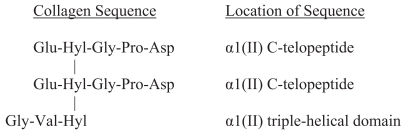
The sequence of the cross-linked Col2CTx epitope. The crosslinked structure of this telopeptide biomarker, indicative of type II collagen degradation, enables it to survive to urine. The cross-link designated as Hyl-Hyl-Hyl is hydroxylysylpyridinoline.

Eyre was the first to describe cross-links in telopeptides of fibril-forming collagens type I, II and III (Eyre, 1989, Eyre, 1991). The structure of cross-linked peptides that originate from the C-terminal telopeptides of type II collagen is shown in Figure 2. Many variations of this basic structure can be isolated from body fluids. For example, the cross-linked peptides can be longer or shorter by 1–3 amino acid residues, e.g. Glu-Hyl-Gly-Pro-Asp-Pro (EKGPDP) or Glu-Hyl-Gly-Pro-Asp-Pro-Leu (EKGPDPL), or Val-Hyl (VK). The hydroxyl group of the 3-hydroxypyridinium cross-link can also be glycosylated. Col2CTx is a composite term given by Eyre, et al. to describe several protease-generated neoepitopes originating from the C-telopeptides of the *α*1 chains of type II collagen fibrils. It is assumed that the cross-linked peptide originating from the triple helical domain is contributed from a different molecule of collagen from that of the telopeptides (which can hypothetically originate from two *α* chains of one molecule). The cross-link probably explains the stability and survival of the epitope from cartilage via blood to urine. Eyre’s group has developed several mAbs to these peptide epitopes (Atley et al. 1998) including mAb 10F2 (Fernandes et al. 2003), and mAb 2B4 (Eyre et al. 2004), enabling these cross-linked telopeptides to be detected by ELISA in body fluids (Eyre, 1995).

Only investigative data are available on mAb 10F2 (Fernandes et al. 2003). Synovial fluid Col2CTx by ELISA (monoclonal antibody 2B4) correlates with the severity of histological OA in the canine (Matyas et al. 2004) and rabbit (Lindhorst et al. 2005) meniscectomy models of OA. ELISA with mAb 2B4 has also been used to analyze sera in familial arthritides (Moskowitz et al. 1998; Christgau et al. 2001), and has demonstrated high levels of Col2CTx in synovial fluid of OA patients and patients after joint injury (Lohmander et al. 2003). Mean (SD) Col2CTx levels by 2B4 ELISA were also higher in patients with generalized knee and hand OA (51.9 ± 20.7 ng/mg Cr), than in patients with single joint knee OA (36.6 ± 8.8 ng/mg Cr), which in turn was higher than levels in healthy controls (42.4 ± 15.2 ng/mg Cr), thus supporting a role as a burden of disease marker (Atley et al. 2000). Amino-terminal telopeptides of type II collagen (Col2NTx) have not been found in body fluids, suggesting they are degraded in vivo all the way to free hydroxylysylpyridinoline.

CTX-II is identical to neoepitope Col2CTx described above, found at the C-terminus of the ¼ length fragment of cleaved type II collagen. It is measured with commercially available kits (Nordic Bioscience, Herlev, Denmark) by ELISA in urine under the name Urine CartiLaps (Christgau et al. 2001), and more recently in sera. The urine and serum assays may potentially be measuring slightly different biochemical epitopes. The urine assay is a competitive ELISA which likely detects monomeric and dimeric CTX-II epitopes, while the serum assay is based on binding of two identical monoclonal antibodies in a sandwich ELISA which likely detects solely the cross-linked dimeric CTX-II (Christgau et al. 2001). The monoclonal antibodies (F4601 and F2603) forming the basis of these assays rely upon the C-terminal proline of the target sequence (EKGPDP) for immunoreactivity (Christgau et al. 2001; Oestergaard et al. 2006). Normal urinary values differ significantly by age and gender, with mean urinary values as follows (ng/mmol creatinine): females 299; peri-premenopausal females 168; peri-postmenopausal females 318; males 278 (Haima, 2005; Mouritzen et al. 2003). This assay probably has the most abundant data supporting its use as an arthritis biomarker. This epitope is elevated: in vitro in human OA explant cultures (Roy-Beaudry et al. 2003); in vivo in the serum in the rat collagen-induced arthritis model (Ishikawa et al. 2004; De Ceuninck et al. 2003), in the synovial fluid of the rat collagen-induced arthritis model (Oestergaard et al. 2006); and in the serum and synovial fluid of rats after intra-articular monoidoacetate injection (Oestergaard et al. 2006). The study by De Ceuninck, et al. also showed that CTX-II responded to collagenase inhibition (De Ceuninck et al. 2003). In addition to this investigational work, Jung, et al. showed elevated uCTX-II in both RA and OA human subjects compared to control. In this study, OA patients had a three-fold higher CTX-II level (527ng/mmol) compared to unaffected controls (190ng/mmol, p < 0.001) suggesting the utility of CTXII as a diagnostic OA biomarker (Jung et al. 2004).

CTX-II may also be useful as a disease burden marker and a prognostic marker. As an indication of degree of cartilage damage, CTX-II has been shown to correlate with degree of joint destruction (Christgau et al. 2001; Garnero et al. 2003; Garnero et al. 2001). Reijman, et al. showed that higher baseline CTX-II level correlated with risk of progression over a six year period in a cohort of 237 knee and 123 hip OA subjects (Reijman et al. 2004). Several studies suggest that CTX-II also has value in efficacy of therapy assessments. In a prospective study comparing patients with knee OA flare treated with rofecoxib versus placebo, uCTX-II levels were 18% lower (p = 0.0003) in the treatment group compared to the placebo group, although the possibility cannot be ruled out that the drug decreased renal excretion of CTX-II (Gineyts E, 2004). Similar effects were observed with ibuprofen (Gineyts et al. 2004). Additionally, in contrast to placebo, adalimumab has been shown to decrease uCTX-II levels in patients with RA (17.3% decrease, p < 0.01) (Garnero P, 2004). Finally, Landewe and colleagues demonstrated a decrease in uCTX-II levels in RA patients after 3 months of treatment with disease modifying anti-rheumatic drug (DMARD) therapy. In this study, combination DMARD therapy with prednisone, sulfasalazine, and methotrexate decreased uCTX-II by 36% compared to 17% for sulfasalazine alone over 3 months. This decline in uCTX-II at 3 months predicted long term (5 year) improvement in radiographic outcome (Landewe et al. 2004).

### Collagen Synthesis Biomarkers

Damage to cartilage also causes the chondrocyte to produce new type II collagen. This is secreted as a procollagen molecule that must undergo post-translational modification. As described above, the released amino- and carboxy-terminal propeptide protein fragments can be used to measure collagen synthesis (Hotta et al. 2005; Shinmei et al. 1993; Rousseau et al. 2004a).

#### CPII

CPII is measured with a commercially available ELISA (IBEX, Montreal, Quebec), and in other formats (Shinmei et al. 1993) and reflects carboxy-terminal type II collagen propeptides in serum, synovial fluid, and cartilage extracts. The CPII assay correlates directly with collagen synthesis (Nelson et al. 1998). Reported normal mean (SD) values for women are 217 ± 60 ng/ml (Haima, 2005). CPII varies significantly by gender with men having higher values than women (mean lnCPII 5.49 in men, 5.36 in women) (Jordan et al. 2004a). CPII immunoreactivity of OA cartilage is markedly elevated (7.6-fold) although this does not appear to be reflected in an increase of this epitope in the serum of OA patients (Nelson et al. 1998). However, in RA sera, CPII is elevated in both rapidly and slowly progressive disease (Mansson et al. 1995). The elevation of serum CPII found in RA subjects could help differentiate RA from OA (Mansson et al. 1995). Using a sandwich ELISA format based on polyclonal antibodies, this epitope has been demonstrated to be elevated in the synovial fluid in patients with OA, RA, and traumatic arthritis (Shinmei et al. 1993), and in the synovial fluid of individuals with varus knee alignment or obesity, suggesting that mechanical stress stimulates chondrocytes to increase collagen synthesis (Kobayashi et al. 2000). The authors concluded that CPII level in synovial fluid is a marker of early OA due to mechanical risk factors. Additional studies support the notion that CPII is elevated in relation to body mass index and mechanical stress (Kobayashi et al. 1997; Kobayashi et al. 2002). Taken together, these data suggest that CPII may be useful for detecting early alterations of cartilage metabolism and cartilage damage.

Synovial fluid CPII may also be useful for determining disease stage and those at risk for progression of OA. In a study by Lohmander et al. CPII levels were compared among healthy controls, knee injury subjects, and those with post-traumatic knee OA (Lohmander et al. 1996). In this study, levels of CPII were elevated shortly after knee injury and peaked within 1–4 years. Levels were 2–4 times higher in the OA and injury groups compared with control. Importantly, levels peaked well before radiographic OA was noted. Only in patients with advanced disease did the CPII levels begin to drop. Correlation of CPII levels with severity of OA suggests its potential as a burden of disease marker (Kobayashi et al. 1997). Synovial fluid concentrations of the same collagen C-propeptide fragment in another commercially available format (Teizin KK, Osaka, Japan), called PIICP, has been shown to be predictive of radiographic knee OA in a four year prospective study(Sugiyama et al. 2003). In a study of ankylosing spondylitis patients, CPII levels were found to be elevated and declined in response to infliximab treatment, suggesting that CPII may be useful as an efficacy of intervention biomarker (Kim, 2004; Kim et al. 2005).

#### PIIANP

Type IIA procollagen contains an additional 69 amino acid cysteine-rich domain in the N-propeptide that binds bone morphogenetic protein 2 and that has been hypothesized to play a role in chondrogenesis (Zhu et al. 1999). Antisera to this sequence were initially developed to localize type IIA procollagen in embryonic tissues (Oganesian et al. 1997). This reagent has been made available in a commercially available ELISA (Linco Research, St Charles, MO) to detect the N-terminal cleavage product of procollagen IIA in sera. PIIANP is indicative of type II collagen synthesis (Rousseau et al. 2004a). As a diagnostic marker, serum PIIANP is decreased in OA patients compared to controls, as was also noted with CPII (Garnero et al. 2002, Rousseau et al. 2004b; Rousseau et al. 2004a). Serum levels of PIIANP are lower in OA compared with RA patients. No data are available on the utility of PIIANP as a marker of disease burden. One study comparing OA to RA suggested that prednisone therapy in RA increased PIIANP levels compared to non-treated controls (15.0 ± 2.4 versus 13.5 ± 2.4 ng/ml, P < 0.05) (Rousseau et al. 2004b). This suggests the possible utility as an efficacy of intervention biomarker.

As noted previously, the use of a combination of biomarkers is a promising approach to the diagnosis, prediction of progression, and response to therapy, of various forms of arthritis. For instance, PIIANP has been combined with CTX-II to predict progression of radiographic knee OA (Garnero et al. 2002). An uncoupling index (decreased PIIANP and increased CTX-II) provided the greatest discriminatory capacity between OA and controls. Subjects with higher baseline uncoupling indices were more likely to have radiographic progression and pain one year later. The combination of low PIIANP (more than one SD below the mean of the controls), and a high CTX-II (more than one SD above the mean of the controls) was associated with an 8-fold more rapid progression of radiographic joint damage. The authors concluded that the combination of these markers was useful for detecting knee OA patients at highest risk for rapid progression.

## Conclusions

Although much progress has been made both developing and validating type II collagen neoepitopes for arthritis applications, many unanswered questions remain. Do different assays in a category measure the same biological process or do they differ in their ability to correlate with incident or progressive joint disease? What threshold level of collagen degradation or synthesis in a particular joint is required to impact serum or urine concentrations? Since joints vary dramatically in size and rates of cartilage metabolism, how much epitope is contributed by a particular joint to body fluids? What are the clearance rates of these collagen biomarkers from joints and the systemic circulation? Do anti-arthritic drugs alter renal or hepatic clearance of a marker and thus confound interpretation of drug effects on joint tissue metabolism? Do subtle variations of an epitope (e.g. post-translational modifications, differences in the length or amino acid content) lead to a difference in what is measured in the serum compared to the urine that can provide meaningful clinical insights? Additional problems arise when considering how to evaluate the ability of a biomarker to predict a meaningful clinical outcome. For example, what “gold standard” endpoint is to be used, radiographic change (plain films, magnetic resonance imaging, other imaging modality) or patient symptoms, to evaluate the performance of the biomarker? The limitations of the so-called “gold standard” outcomes are well known and are part of the motivation to develop molecular biomarkers in the first place.

It is also very important that additional efforts are made to better understand what exact biochemical species we are measuring in complex biological fluids such as urine, serum and synovial fluid. Assays that work very well under defined laboratory conditions may lose some or all of their specificity when attempting to measure very low levels of specific epitopes in complex biological backgrounds. It is also important to understand potential sequence differences in species that may lead to variations in affinity and specificity of a particular assay. Newer methods in use and development, for instance the uTIINE assay, that involves immunocapture followed by mass spectroscopy, may provide more precise quantification of a specific epitope.

Despite these many questions and cautionary notes, it is apparent that numerous useful and promising biomarkers, in various stages of development, are currently available, even just considering this single protein, type II collagen. Since individual biomarkers have various limitations, it is likely that combining biomarkers will be the most effective means of diagnosing arthritis early, as well as to assess burden of disease, determine efficacy of therapy, and predict which patients will have progressive disease. It seems probable that a clinical profile will be assessed in future that includes family history, body habitus, joint injury, patient symptoms, radiographs, and biomarkers, to determine a patients risk profile and help the physician diagnose and manage arthritis at the earliest stage possible.

## Figures and Tables

**Figure 1a:**
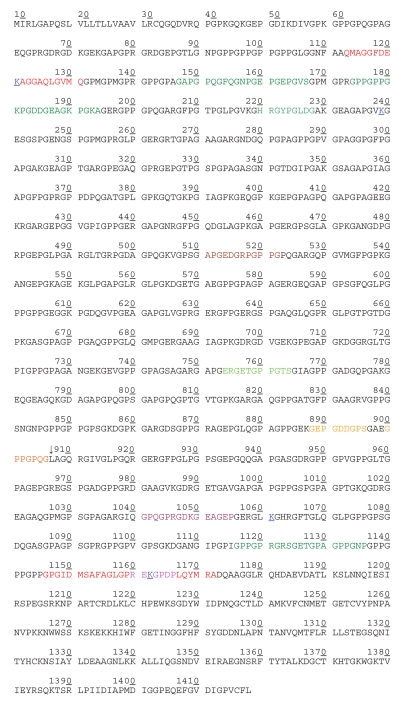
Amino acid sequence in *α*1(II) chain of human procollagen type IIB (COL2A1_HUMAN, P02458, UniProtKB/Swiss). Numbering of amino acids in this figure (and throughout the whole text of this review) is consistent with numbering in the source given above and may not correspond to numbering in a particular reference. Numbering used here includes N-terminal signal peptide and N-propeptide, and does not include the alternatively spliced block of 69 amino acids in the N-propeptide (which is shown in Figure 1b). Each number corresponds to the last one of ten amino acids under it. - Tandems Proline/Hydroxyproline and Lysine/Hydroxylysine are not distinguished and are represented by the same letter (P and K, respectively). - Positions of cross-links are color-coded and underlined (K^121, 239, 1061, and 1162^). - The telopeptides (in red lettering) and epitopes discussed in this review is as follows: 113–131 N-terminal non-helical domain (N-terminal telopeptide) 1146–1172 C-terminal non-helical domain (C-terminal telopeptide) 132–1145 Triple-helical domain 1173–1418 Carboxy-terminal propeptide domain 26–112 Amino-terminal propeptide domain

**Figure 1b:**
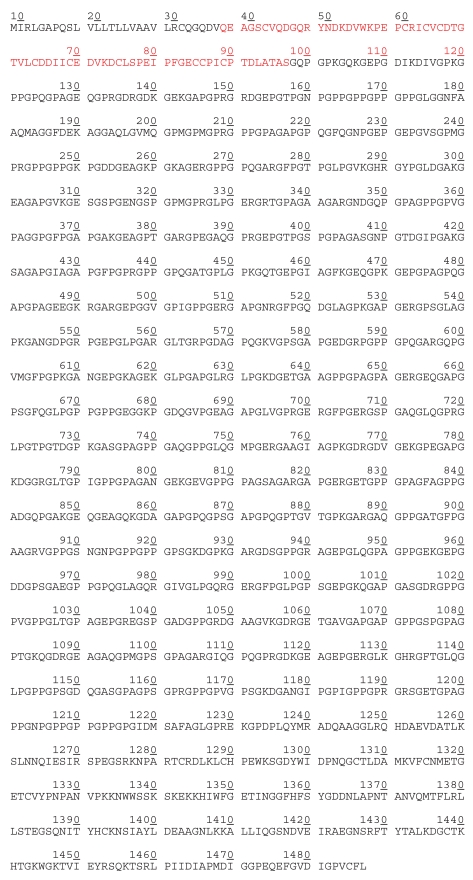
Amino acid sequence of *α*1(II) chain of human procollagen type IIA (UniProtKB/TrEMBL entry Q14047) 29–97 The alternatively spliced 69 amino acids of the amino-terminal propeptide domain.

**Table 1:** Potential Arthritis Biomarkers.

Type II Collagen Related Epitopes (see Table 2)
Aggrecan Related Epitopes Chondroitin sulfate epitopes 2B6, 3B3 and 846 Keratan Sulfate Uronic acid Total glycosaminoglycan (dimethylmethylene blue binding)
Hyaluronan
Link Protein
Cartilage Oligomeric Matrix Protein
Cartilage Intermediate Layer Protein
Proline Arginine-Rich End Leucine-Rich Protein
Decorin
Matrix Metalloproteinases
Cytokine Profiles
C-Reactive Protein
Fibromodulin
Fibronectin Fragments
Human Cartilage Glycoprotein 39 (YKL-40)
Glucosyl-Galactosyl-Pyridinoline
Peripheral Blood Mononuclear Cell RNA ExpressionProfiles
Bone Turnover Biomarkers NTXI CTXI

**Table 2:** Currently available biomarkers of type II collagen degradation and synthesis.

Biomarker Name^a^	AA Sequence	AA Residues^b^	Specificity^c^	Body Fluid or Tissue^d^	Ab^e^	BIPED Classification^f^	References Related to Assay Development
**Collagen Degradation**							
**Cleavage Neoepitopes**							
Col2-1/4N1	LAGQRG	907–912	Not specified	C	p(Fab)_2_	I	(Billinghurst et al., 1997)
Col2-1/4N2	QRGIVG	910–915	CII	C	p(Fab)_2_	I	(Billinghurst et al., 1997)
C1,2C (COL2-3/4C_short_)	GPP(OH)GPQG	899–906	CI and CII	S, SF, C	p	P (combined with C2C or CPII), D	(Billinghurst et al., 1997)
C2C (COL2-3/4C_long mono_)	E GPP(OH)GPQG	898–906	CII in humans	S, SF, U	m	P (combined with C1,2C Or CPII), E, D	(Poole et al., 2004)
uTIINE (5109 and 9A4 ELISA; 5109 capture in LC-MS/MS format)	GEPGDDGPS/GPPGPQG;ARGDSGPPGRAGEPGLQGPAGPPGEKGEPGDDGPSGAEGPPGPQG	888–896/899–906; 862–906	CII	U	m/m; m/LC-MS/MS	B, P, E, D	(Otterness et al., 1999, Downs et al., 2001, Burgeson and Nimni, 1992, Hellio Le Graverand et al., 2006)
**Denaturation Neoepitopes**							
COL2-3/4m	APGEDGRPGPPG	511–522	CII, CXI	C, S	m	D	(Hollander et al., 1994)
Coll 2-1 Coll 2-1 NO_2_	HRGYPGLDG	220–228	CII	S, U	p	P, D	(Deberg et al., 2002, Henrotin et al., 2004)
Helix-II^∞^	ERGETGPP(OH) GTS	754–764	CII	U	p	P, D	(Charni et al., 2005)
AH12	GAPGPQGFQGNPGEPGEPGVS	147–167	CII	C	p	I	(Croucher and Hollander, 1999)
AH8	GPPGPPGKPGDDGEAGKPGKA	174–194	CII	C	m and p	I	(Croucher and Hollander, 1999)
AH9	GPP(OH)GP RGRSGETGPAGPP(OH)GNP(OH)	1116–1136	CII	C	p	I	(Croucher and Hollander, 1999)
CII CNBr9.7 (18:6:D6 and 14:7:D8 ELISA)	Epitope within CNBr 9.7/GPQGPRGDKGEAGEP	1028–1151/1041–1055	CII/CI, CII, CIII, CV	SF	m/m	D	(Barrach et al., 1996, Elsaid and Chichester, 2006)
**Telopeptide Epitopes**							
col2CTx g CTX-II (Urine CartiLaps and preclinical serum assay) g	(R)EKGPDP	1160–1166	CII cross-links	C, S, P(EDTA), SF, U	m, m/m	B, P, E, D	(Eyre, 1991, Christgau et al., 2001, Oestergaard et al., 2006, Matyas et al., 2004, Eyre, 1989)
**Collagen Synthesis**							
CPII PIICP (chondrocalcin)	Sequence within C-propeptide: DQAAGGLR Q…DIGPVCFL	1173–1418 (of procollagen IIB in Figure 1a or 1242–1487 in procollagen IIA in Figure 1b)	CII	S, SF, C	p p/p	B, P, E, D	(Van der Rest et al., 1986, Sugiyama et al., 2003, Mansson et al., 1995, Shinmei et al., 1993)
PIIANP	QEAGSCVQDG QRYNDKDVW KPE PCRICVCDTGT VLCDDIICEDV KDCLSPEIPFG ECCPICPTDLA TAS	29–97 (of procollagen IIA in figure 1b)	CII	S (not plasma)	p	P (combined with uCTXII), E, D	(Oganesian et al., 1997, O’Leary et al., 2004)

a The biomarker name is the usual one in the literature and does not discriminate if it applies to an antibody, an epitope, or an assay name.

b Categories are based on localization of an epitope in the type II collagen molecule and residue numbering is based on the human type II collagen sequence, P024588 in UniProtKB/Swiss sequence shown in Figure 1a:
-Cleavage neoepitopes localized to the collagenase cleavage site between Gly^906^ and Leu^907^ -Denaturation neoepitopes localized to the triple-helical domain -Epitopes localized to the telopeptides -Collagen synthesis epitopes localized to the N-propeptide domain (AA 26–112) or C-propeptide domain (AA 1173–1418).

c CII = type II collagen; CI = type I collagen; CIX = type IX collagen.

d The table shows human body fluid or tissue for which the application of the assay is documented in the literature: S = serum; SF = synovial fluid; U = urine; C = cartilage explant.

e m - monoclonal antibody (mAb), p - polyclonal antiserum, m/m - a sandwich of two mAbs, LC-MS/MS - liquid chromatography with mass spectroscopy.

f Provisional BIPED classification categories based on Bauer et al. (Bauer et al. 2006): B = burden of disease; I = investigative; P = prognostic; E = efficacy of intervention; D = diagnostic marker.

g The antibodies to Col2CTx and CTX-II are specific for a peptide that happens to be present in the cross-linked structure in human body fluids but the cross-link is not a part of the epitope.
